# Impact of Pre-Anthesis Drought Stress on Physiology, Yield-Related Traits, and Drought-Responsive Genes in Green Super Rice

**DOI:** 10.3389/fgene.2022.832542

**Published:** 2022-03-24

**Authors:** Hassaan Ahmad, Syed Adeel Zafar, Muhammad Kashif Naeem, Sajid Shokat, Safeena Inam, Malik Attique ur Rehman, Shahzad Amir Naveed, Jianlong Xu, Zhikang Li, Ghulam Muhammad Ali, Muhammad Ramzan Khan

**Affiliations:** ^1^ National Institute for Genomics and Advanced Biotechnology (NIGAB), National Agricultural Research Centre, Islamabad, Pakistan; ^2^ Nuclear Institute for Agriculture and Biology, Faisalabad, Pakistan; ^3^ Institute of Crop Science, Chinese Academy of Agricultural Sciences, Beijing, China

**Keywords:** drought, anthesis, pollen fertility, grain yield, correlation, drought-responsive genes

## Abstract

Optimum soil water availability is vital for maximum yield production in rice which is challenged by increasing spells of drought. The reproductive stage drought is among the main limiting factors leading to the drastic reduction in grain yield. The objective of this study was to investigate the molecular and morphophysiological responses of pre-anthesis stage drought stress in green super rice. The study assessed the performance of 26 rice lines under irrigated and drought conditions. Irrigated treatment was allowed to grow normally, while drought stress was imposed for 30 days at the pre-anthesis stage. Three important physiological traits including pollen fertility percentage (PFP), cell membrane stability (CMS), and normalized difference vegetative index (NDVI) were recorded at anthesis stage during the last week of drought stress. Agronomic traits of economic importance including grain yield were recorded at maturity stage. The analysis of variance demonstrated significant variation among the genotypes for most of the studied traits. Correlation and principal component analyses demonstrated highly significant associations of particular agronomic traits with grain yield, and genetic diversity among genotypes, respectively. Our study demonstrated a higher drought tolerance potential of GSR lines compared with local cultivars, mainly by higher pollen viability, plant biomass, CMS, and harvest index under drought. In addition, the molecular basis of drought tolerance in GSR lines was related to upregulation of certain drought-responsive genes including *OsSADRI*, *OsDSM1*, *OsDT11*, but not the DREB genes. Our study identified novel drought-responsive genes (*LOC_Os11g36190*, *LOC_Os12g04500*, *LOC_Os12g26290*, and *LOC_Os02g11960*) that could be further characterized using reverse genetics to be utilized in molecular breeding for drought tolerance.

## Introduction

Rice (*Oryza sativa* L.) is one of the primary staple food crops for nearly 50% of the world population (Zafar et al., 2018). The countries located in East Asia, South Asia, and Southeast Asia are dominant in production and consumption of rice across the globe. Historically, more than 90% of world rice production is contributed from these countries (Barker et al., 1999). Its production is needed to increase by 0.6%–0.9% per year until 2050 to feed the further 2 billion people (Desa, 2015). However, different abiotic and biotic stresses are major limiting factors for obtaining higher yield in rice (Zafar et al., 2017; Oliva et al., 2019; Ahmed et al., 2021). Being a water-loving plant, rice is highly sensitive to drought stress, which significantly affects its grain yield (Shuxing, 2014). Drought is becoming a serious yield constraint for various major crops due to global water scarcity (Wattoo et al., 2018; Hussain et al., 2019; Shokat et al., 2020a). A recent study using the yield and metrological data from 1980 to 2015 reported the yield decline up to 21% in wheat (*Triticum aestivum* L.) and 40% in maize (*Zea mays* L.) due to drought on a global scale (Daryanto et al., 2016). In rice, mild-drought stress reduced grain yield by 31%–64%, while severe stress reduced 65%–85% yield compared with normal conditions (Kumar et al., 2008). It affects the yield by altering different agronomic and physiological traits including plant height, number of tillers, leaf area, leaf rolling, transpiration rate, accumulation of osmoprotectants, root system, and stomatal closure (Nakashima et al., 2007; Islam et al., 2009; Tong et al., 2009). Anthesis stage drought stress can interrupt flowering, floret initiation (Bajji et al., 2002), pollen fertility (Zhou et al., 2011), and grain filling, resulting in poor paddy yield. Rice growth is affected by drought at different stages including booting (Shao et al., 2014), flowering (Liu et al., 2006), and grain filling stage (Zhang et al., 2018). However, drought stress at anthesis stage restricts the availability of photosynthates by disturbing the sink capacity (Do et al., 2010) and reduces the grain yield, plant biomass, and ultimately the harvest index (Blum, 2018). It also impairs anther dehiscence, pollen viability, and pollen germination in rice resulting in spikelet sterility and more sterile grains in the panicles (Prasad et al., 2017). Drought induced spikelet sterility is considered as one of the major causes of yield reduction.

To address the challenge, natural variation in rice germplasm for drought tolerance could be exploited to identify the drought-tolerant genotypes, the associated traits, and underlying genes (Panda et al., 2021). In addition, induced variation *via* hybridization and mutagenesis could serve as an important genetic resource for target breeding (Zafar et al., 2020c). For the purpose, scientists have started to put efforts to breed green super rice (GSR), an elite rice type that could withstand multiple stresses with high nutrient-use efficiency (Wing et al., 2018; Jewel et al., 2019). The idea was given by a famous rice geneticist Qifa Zhang in 2007 (Zhang, 2007), which was later implemented by a team of international scientists from China and the International Rice Research Institute (IRRI), Philippines (Yu et al., 2020). The present study was conducted to evaluate 22 selected GSR lines along with four local rice cultivars for drought tolerance in Pakistan, and identify agronomic and physiological traits associated with drought tolerance in GSR. In addition, the contrasting drought tolerant and sensitive lines were assessed for gene expression profile to identify underlying genes related to drought tolerance in GSR. This study identified high-yielding drought-tolerant GSR lines and provided us knowledge about drought tolerance-related traits, and novel drought-related genes.

## Materials and methods

### Experimental site

The field experiment was conducted at the National Institute for Genomics and Advanced Biotechnology, NARC, Pakistan (33.684°N and 73.048°E) during rice growing period (May–October, 2020). To minimize the water infiltration from control to drought plot, a 6- to 8-feet path was made between both plots, and furthermore, plastic film was applied under the soil surface with a depth of 60 cm.

### Experimental design

The 22 diverse GSR lines were selected based on diverse phenotypic characteristics from the 552 GSR genotypes (Supplementary Table S1). Twenty-two GSR lines along with four checks were evaluated using split plot randomized complete block design with two treatments (well-watered and drought) each having three replications. Seeds were sown in nursery trays and 30-day-old seedlings were transplanted in the field. Each plot consisted of five rows of 10 plants with 30-cm row/row and plant/plant distance (Yugandhar et al., 2017). Both plots were irrigated normally (8–10 cm) until anthesis stage. Fertilizer, weedicide, and insecticide application was done according to recommended dosage. Crop cultivation was carried out according to normal cultural practices.

Drought was imposed for 30 days by withholding the applied water at the beginning of anthesis stage. Physiological traits were recorded during the last week of stress. After 30 days, the field was rewatered. At physiological maturity, five representative plants were selected for the measurement of agronomic traits from the three middle rows of each replication to avoid confounding border effects (Chaturvedi et al., 2017).

### Physiological measurements

#### Cell membrane stability

Leaf samples were collected at the last week of drought stress to examine the cell membrane stability by recording the electrolyte conductivity using and electrical conductivity meter (HI 9811-5 Portable EC meter HANNA^®^ Instruments, USA). Flag leaves from three plants per replicate (of each genotype) were collected from both control and drought stress fields in 20-ml glass vials. Further measurement was recorded as proposed by Tripathy et al. (2000). CMS was formulated as the reciprocal of cell membrane injury by using the following formula (Blum and Ebercon, 1981):

#### CMS% = {[1 − (T1 / T2)] / [1−(C1 / C2)]} × 100

where, T and C refer to stressed and controlled, respectively. C1 (initial control), T1 (initial stress), and after autoclave, C2 (final control), T2 (final stress) were the assumed conductance.

#### Normalized difference vegetation index

Normalized difference vegetation index (NDVI) is a spectral reflectance-based measure of the density of green vegetation on a land area. NDVI measurements were taken using GreenSeeker™ Handheld Optical Sensor Unit (NTech Industries, Inc., USA), keeping the sensor at 0.5–1 m above the central rows of all the genotypes individually in three replications of both control and stress field plots (Govaerts and Verhulst, 2010).

#### Pollen fertility test

About five to eight mature spikelets from five panicles (one from each plant) were collected in the morning before anthesis. Spikelets were fixed in FAA solution (formaldehyde:ethanol:acetic acid with a ratio of 1:18:1, respectively) until staining. Anthers were crushed with forceps on glass slide to release pollens, which were immersed in 1% potassium iodide (I2-KI) solution followed by observation under a light microscope (NIKON DIGITAL SIGHT DS-Fi2). Pollens that stained black and circular were considered fertile, while those stained red-orange and of irregular shape were considered sterile (Zafar et al., 2020b). Pollen fertility percentage (PFP) was calculated using the following formula:

ΡFΡ=Number of fertile pollens/ total number of pollens × 100

### Measurement of agronomic traits

Agronomic traits, including plant height per plant (PH), tillers per plant (TPP), grain yield per plant (GY), straw yield per plant (SY), total biomass per plant (TBM), 1,000-grain weight (TGW), and grain length (GL) were recorded manually. Harvest index (HI) was calculated as the ratio of GY to TBM. Drought susceptibility index (DSI) was calculated as [(1−Y / YP) / D] as described earlier (Khanna-Chopra and Viswanathan, 1999; Zafar et al., 2020a). Here, Y is the grain yield under stress conditions, and YP is the grain yield under normal conditions, while D represents the stress intensity, which was calculated as D = (1 − X / XP), where X and XP are means of Y and YP, respectively. Measurements for these traits were carried out on five randomly selected plants of each genotype from each replication by following the method (IRRI, 2002).

### RNA extraction and cDNA synthesis

Total RNA was extracted from the panicles of selected drought-tolerant and -sensitive genotypes from both well-watered (WW) and drought-stressed plants. Panicles were harvested from plants and immediately kept in liquid nitrogen followed by storage at −80°C to avoid the denaturation of RNA. The PureLink RNA Mini kit (Thermo Fisher Scientific) was used to extract the total RNA, in accordance with the manufacturer’s protocol. The quality of isolated RNA was observed on 1.5% RNase-free agarose gel and quantified using the BioSpec-nano spectrophotometer. One microgram of total RNA was used to reverse transcribe into cDNA using RevertAid Reverse Transcriptase kit (Thermo-Fisher Scientific) following the manufacturer’s instructions.

### Differentially expressed gene selection and quantitative real-time PCR

Ten differentially expressed genes (DEGs) under drought stress were selected from a comparative transcriptome study in rice (Huang et al., 2014). To our knowledge, these genes have not been studied before specifically for drought response. In addition, we studied the expressions of three known drought tolerance-related genes: *Oryza sativa Salt-*, *ABA- and Drought-Induced RING Finger Protein 1* (*OsSADR1*) (Park et al., 2018), *Drought-Hypersensitive Mutant1* (*DSM1*) (Ning et al., 2009), *Drought tolerance 11* (*OsDT11*) (Li et al., 2017), *OsDREB1E*, and *OsDREB2B* (Chen et al., 2008). Selected genes are listed in Supplementary Table S2. Coding sequences (CDS) of the selected DEGs were retrieved from the Rice Genome Annotation project (http://rice.plantbiology.msu.edu/cgi-bin/gbrowse/rice/). A gene-specific pair of primers was designed using AmplifX version 1.7.0 software, and primer sequences are listed in Supplementary Table S3.

Quantitative real-time PCR (qRT-PCR) was carried out to determine the relative expression levels of 13 selected genes on StepOne™ Real-Time PCR System (Thermo Fisher Scientific) using Maxima SYBR Green. The delta cT method was used to calculate the relative expression level of each gene, and rice *Actin1* gene was used to normalize the expression (Fang et al., 2021; Zafar et al., 2021).

### Statistical analysis

Morphophysiological traits data were analyzed by analysis of variance using SPSS software according to split plot randomized complete block design. Principal component analysis (PCA) was done through the XL-STAT software (ver. 2018) to categorize various physiological and morphological traits (Mohammadi and Prasanna, 2003). Pearson’s correlation matrix analysis was done using the “cor” package in R studio. The *p*-values for the coefficient of correlation (r) were obtained by applying Student’s *t*-test with the “cor.test” function in R-studio. In the correlation matrix plot, only significant relationships were labeled with stars. Expression pattern significance was calculated using *t*-test*.*

## Results

### Analysis of variance showed significant variation among green super rice accessions under drought stress

Analysis of variance (ANOVA) was performed to see the significant differences of variation among the genotypes and water treatments for physiological and yield-related traits. ANOVA showed significant variation (*p *< 0.01) among the tested genotypes for PH, GY, HI, TGW, GL, and NDVI (Table 1), while nonsignificant differences were observed for TPP, SY, and PFP. There was no significant effect on the studied traits among the replications, which strengthen the reliability of this experiment. Drought significantly affected PH, GY, SY, HI, GL, PFP, and NDVI, while traits, such as TPP, TBM, and TGW, were not affected by drought. The genotype × environment interaction was also significant for PH, TPP, SY, HI, TGW, PFP, and NDVI (Table 1) where pronounced reduction was recorded under drought conditions. Since PH, GY, HI, GL, and NDVI displayed significant differences for genotypes as well as drought treatment, these traits could be key selection markers for drought tolerance screening in rice.

**TABLE 1:** Mean square values from the analysis of variance for the effect of genotype, environment, and their interaction on agronomic and physiological traits.

Source of variation	DF	Plant height (PH)	Tillers per plant (TPP)	Grain yield (GY)	Straw yield (SY)	Total biomass (TBM) (ns)	Harvest index (HI)	1,000 grain weight (TGW)	Grain length (GL)	Pollen fertility percentage (PFP)	Nomralized difference vegetative index (NDVI)
Genotype	25	687.55**	47.97 ^ns^	956.25**	599.24 ^ns^	2,136.97	0.0081*	42.40**	4.02**	727.03 ^ns^	0.0042**
Replication	2	42.40 ^ns^	107.97 ^ns^	700.96 ^ns^	875.40 ^ns^	3,633.82	0.00056 ^ns^	8.87 ^ns^	0.38 ^ns^	15.07 ^ns^	0.02 ^ns^
Treatment	1	1,243.68**	0.27 ^ns^	19,649.7*	5,543.01*	2,969.05	0.36**	106.91 ^ns^	6.31**	5,434.18*	0.14**
Genotype × treatment	25	57.59**	37.34**	236.93 ^ns^	520.24*	1,029.61	0.0033**	3.72**	0.20 ^ns^	771.38**	0.0013*

Significance levels are indicated: *p < 0.05; **p < 0.01. df, degrees of freedom; PH, plant height; TTP, tillers per plant; GY, grain yield; SY, straw yield; TBM, total biomass; HI, harvest index; TGW, thousand-grain weight; GL, grain length; PFP, panicle fertility percentage; NDVI, normalized difference vegetation index.

### Principal component analysis revealed genetic variation among green super rice accessions under drought

**FIGURE 1:**
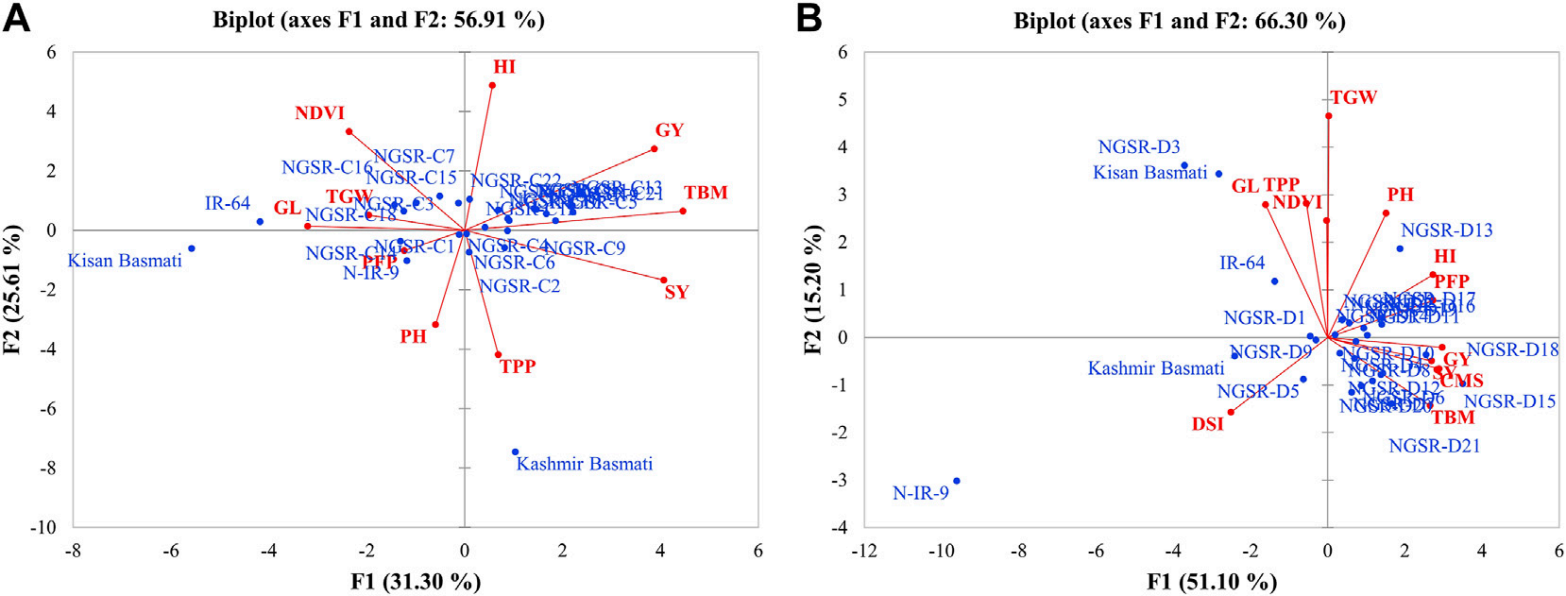
PCA showing biplot for genotypes and studied traits **(A)** under normal condition and **(B)** under drought stress condition.

Separated PCA analyses were performed to develop a trait–genotype (T–G) biplot and to detect genetic variation among the studied genotypes for various morphophysiological traits under well-watered and drought-stressed conditions. Under WW environment, a biplot was drawn between PC1 and PC2 explaining 31.3% and 25.6%, of total variation, respectively (Figure 1A). Our results indicate that SY, GY, HI, and TBM were in the opposite direction of NDVI, TGW, and GL indicating their opposite relationship with each other. In addition, the GSR lines were mostly clustered near the origin and show less genetic variability, while checks Kashmir Basmati, Kissan Basmati, and IR-64 were widely distributed apart from the origin and showed remarkable genetic variability (Figure 1A). In case of drought treatment, the PC1 alone accounted for 51.10% of the total variability, while PC2 shared 15.20% (Figure 1B). Results of this experiment show GY were clustered closer to PFP, CMS, TBM, and HI, while it was in opposite direction of NDVI and DSI. In contrast with the WW treatment, many GSR lines, namely, NGSR-3, NGSR-15, NGSR-18, NGSR-13, NGSR-21, and NIAB-IR-9, fall near the apex of the biplot and show remarkable genetic variation under drought stress (Figure 1B). The check varieties Kissan Basmati, NIAB-IR-9, and Kashmir Basmati also showed considerable genetic variability and reputation of these accessions for further selection in breeding programs.

### Mean performance of green super rice accessions for studied traits

**FIGURE 2:**
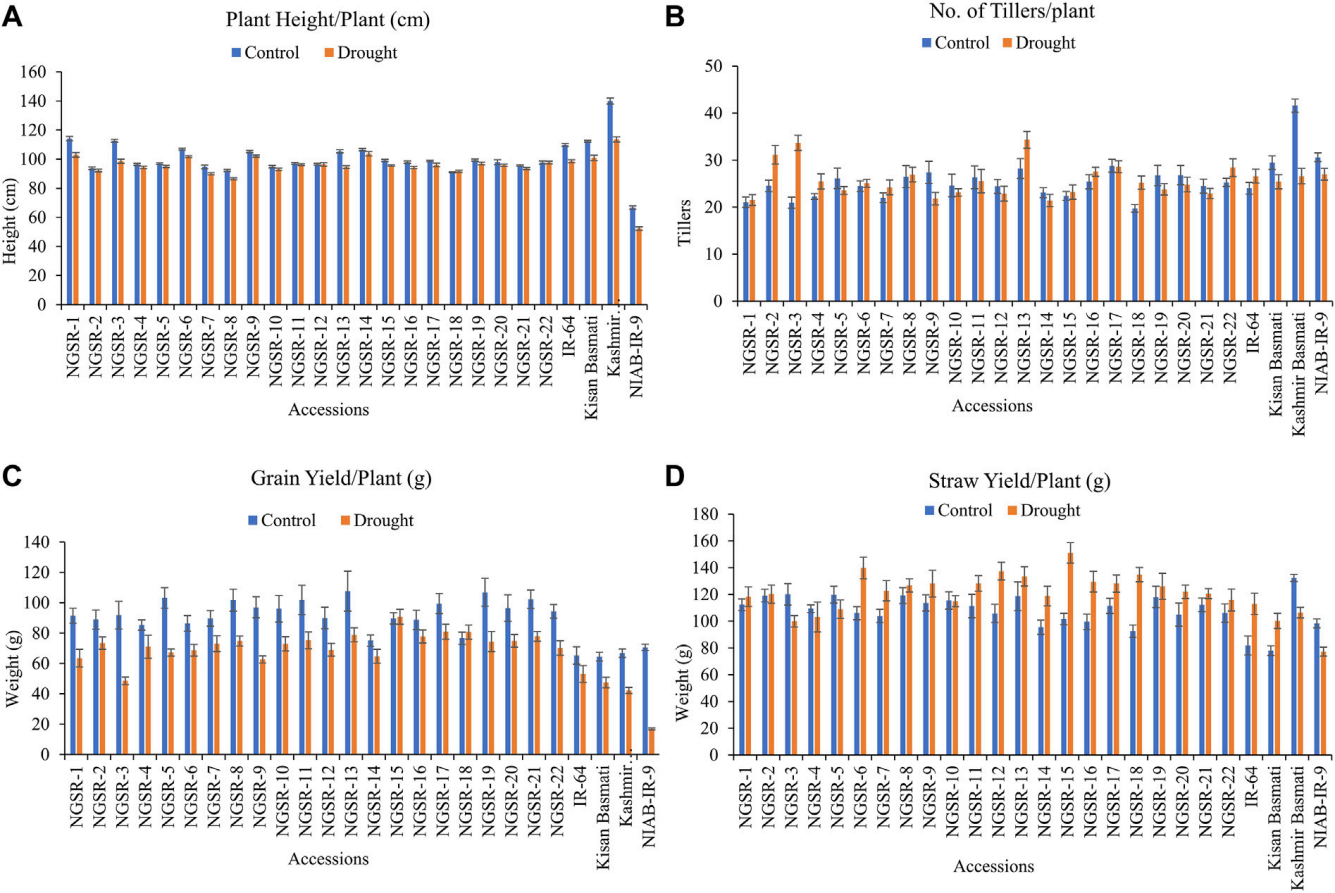
Effect of drought stress on **(A)** plant height/plant, **(B)** tillers/plant, **(C)** grain yield/plant, and **(D)** straw yield/plant. Values are means ± SD.

**FIGURE 3:**
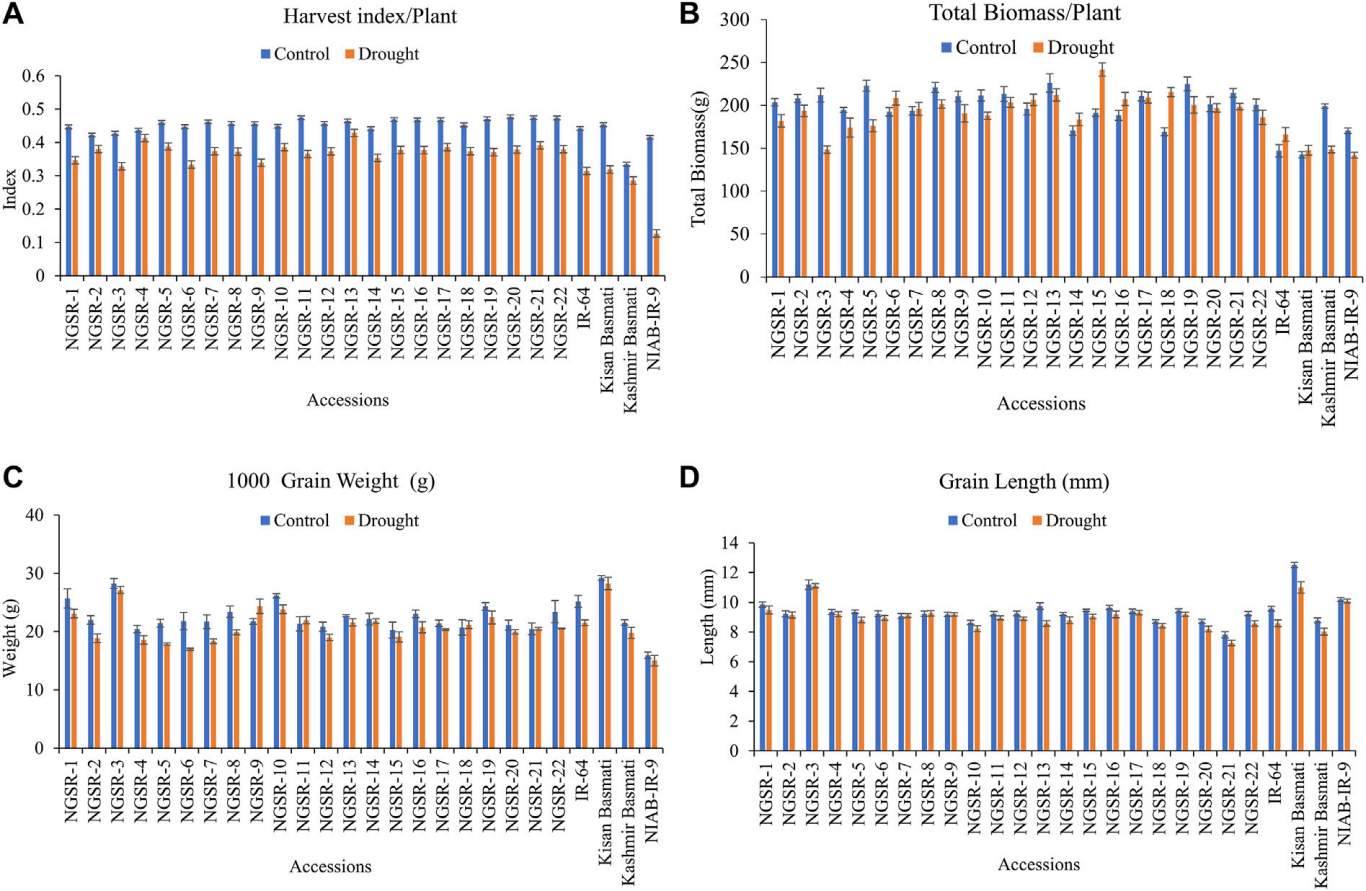
Effect of drought stress on **(A)** harvest index/plant, **(B)** total biomass/plant, **(C)** 1,000-grain weight, and **(D)** grain length. Values are means ± SD.

Drought stress showed a remarkable reduction in grain yield and yield-related traits in all studied genotypes except NGSR-15 and NGSR-18 (Figures 2 and 3). Among the 22 GSR lines, the minimum PH (86.6 cm) under drought condition was attained by NGSR-8 and the maximum (103.7 cm) was recorded by NGSR-14, whereas among the four checks, the maximum PH was recorded for Kashmir Basmati (113.6 cm), and the minimum was depicted by NIAB-IR-9 (52.3 cm). All GSR lines demonstrated higher PH than the drought-sensitive check NIAB-IR-9 (Figure 2A). These results suggested that the GSR lines were comparatively less affected by drought stress and maintained the normal plant growth.

Overall, TPP were not significantly affected under drought stress except for Kashmir Basmati, while a few of the GSR lines showed increased TPP under drought (Figure 2B). GY is the most important agronomic trait of economic importance, and drought stress affected the GY in most genotypes except NGSR-15 and NGSR-18 (Figure 2C). Under drought stress, maximum GY was reported by NGSR-15 (90.7 g) and the minimum by NGSR-3 (48.6 g). Among the checks, IR-64 showed the maximum GY^−1^ (53.1 g), whereas the minimum was shown by the drought-sensitive NIAB-IR-9 (16.8 g). All the GSR lines (except NGSR-3) demonstrated higher grain yield than the check varieties; even the drought-sensitive NGSR-3 accounted for higher grain yield than the sensitive check NIAB-IR-9 (Figure 2C). These results suggested that the grain yield of GSR lines was less affected by drought stress compared with local checks.

SY was generally increased in most GSR accessions along with two check varieties IR-64 and Kissan Basmati under drought stress (Figure 2D). The maximum increase in SY was observed in NGSR-15, NGSR-6, NGSR18, and NGSR-12. However, NGSR-3, Kashmir Basmati, and NIAB-IR-9 showed a decrease in SY under drought. It is noteworthy that SY was only decreased in the most drought-sensitive GSR line and checks, thus, it is considered an important trait for drought escape at the flowering stage. This is because plants tend to continue their vegetative stage bypassing the flowering stage until they got favorable conditions.

Generally, drought stress negatively impacted the HI in all genotypes, but the non-GSR lines showed a higher decrease compared with the GSR lines with the highest decrease observed in our drought-sensitive check NIAB-IR-9 (Figure 3A). These results suggest that GSR lines have the potential to maintain the HI under drought stress conditions (Figure 2A).

TBM was not significantly affected under drought stress except for Kashmir Basmati and NIAB-IR-9 (Figure 3B). Similarly, TGW was not significantly affected under drought stress in the tested genotypes (Figure 3C).

Drought stress had a significant effect on GL; however, differences for genotypes were nonsignificant (Figure 3D). Three genotypes, including NGSR-3, NGSR-1, and NGSR-15, showed longer GLs. The genotypes NGSR-3 and NGSR-21 showed the highest (11.1 mm) and the lowest (7.3 mm) GL, respectively. Among the experimental checks, the maximum (10.9 mm) and the minimum (8.03 mm) GLs were depicted by Kissan Basmati and Kashmir Basmati, respectively. Overall, GSR lines maintained the grain length under drought stress compared with sensitive checks (Figure 3D), except NGSR-21, which showed a reduced grain length (7.3 mm).

### Drought susceptibility index

**FIGURE 4:**
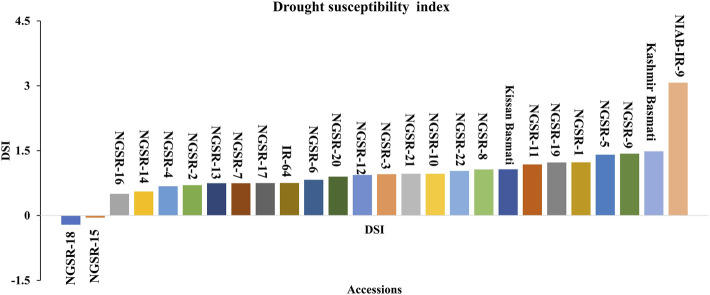
Frequency distribution of drought susceptibility index for grain yield showing the degree of susceptibility to drought stress. Genotypes below the line are declared as most drought-tolerant genotypes.

DSI is an important indicator of drought tolerance, and a lower value indicates better tolerance. Overall, the GSR lines showed lower DSI compared with local varieties, and the genotypes NGSR-18, NGSR-15, and NGSR-16 exhibited the lowest DSIs among 22 GSR lines, showing their potential toward drought tolerance (Figure 4). In contrast, the highest DSI was recorded for NIAB-IR-9 followed by Kashmir Basmati, indicating the least drought tolerance among the tested genotypes. Overall, these results demonstrated that GSR lines generally performed better than the checks.

### Pollen fertility percentage

**FIGURE 5:**
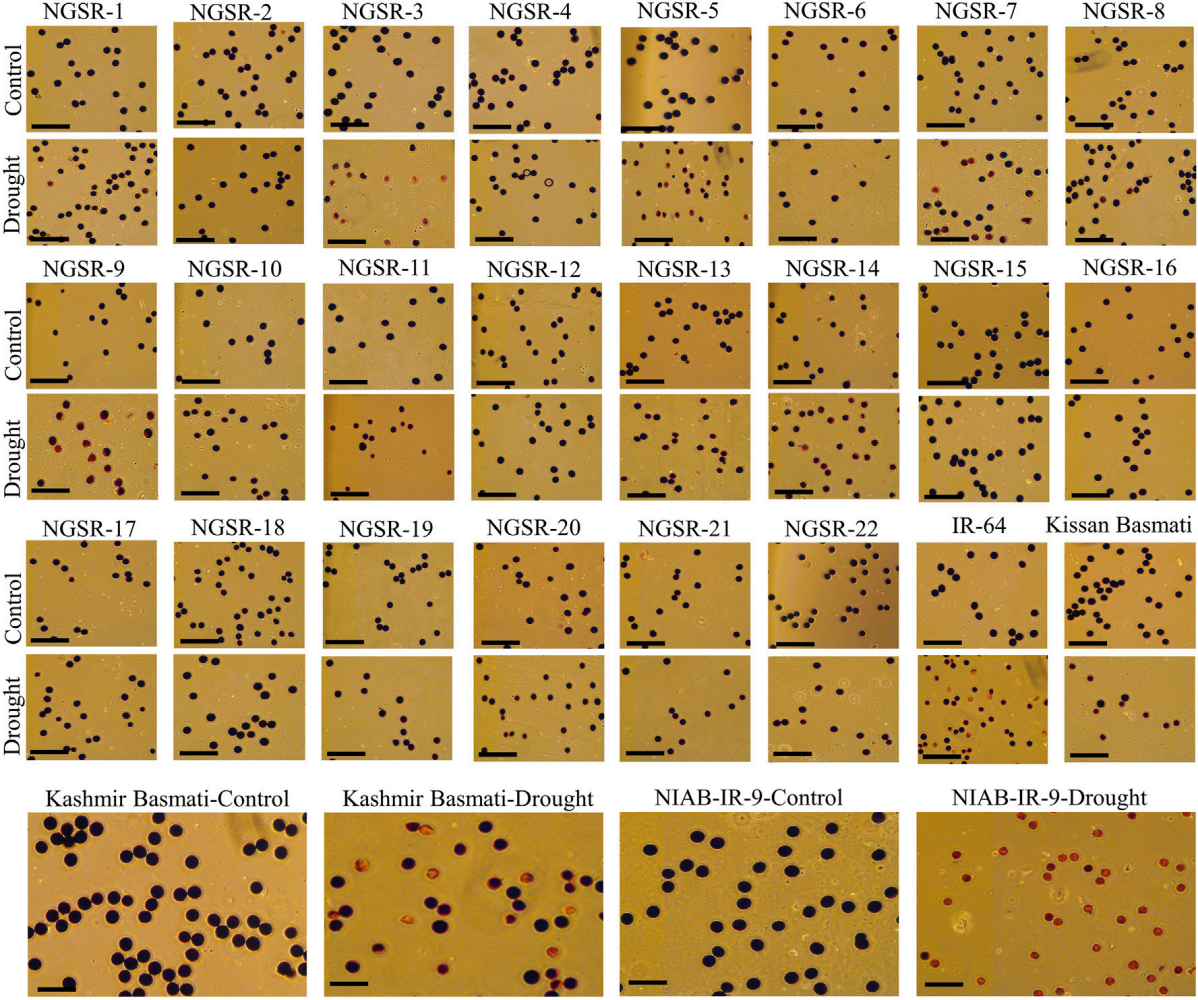
Examination of pollen fertility of the 22 GSR lines and 4 checks with I_2_-KI solution staining of the mature pollen grains. The sterile pollen grains failed to be stained or stained weakly, indicating that they did not contain starch or contained irregularly distributed starch, whereas the viable pollen grains were stained deep brown. Scale bars are 100 µm.

**FIGURE 6:**
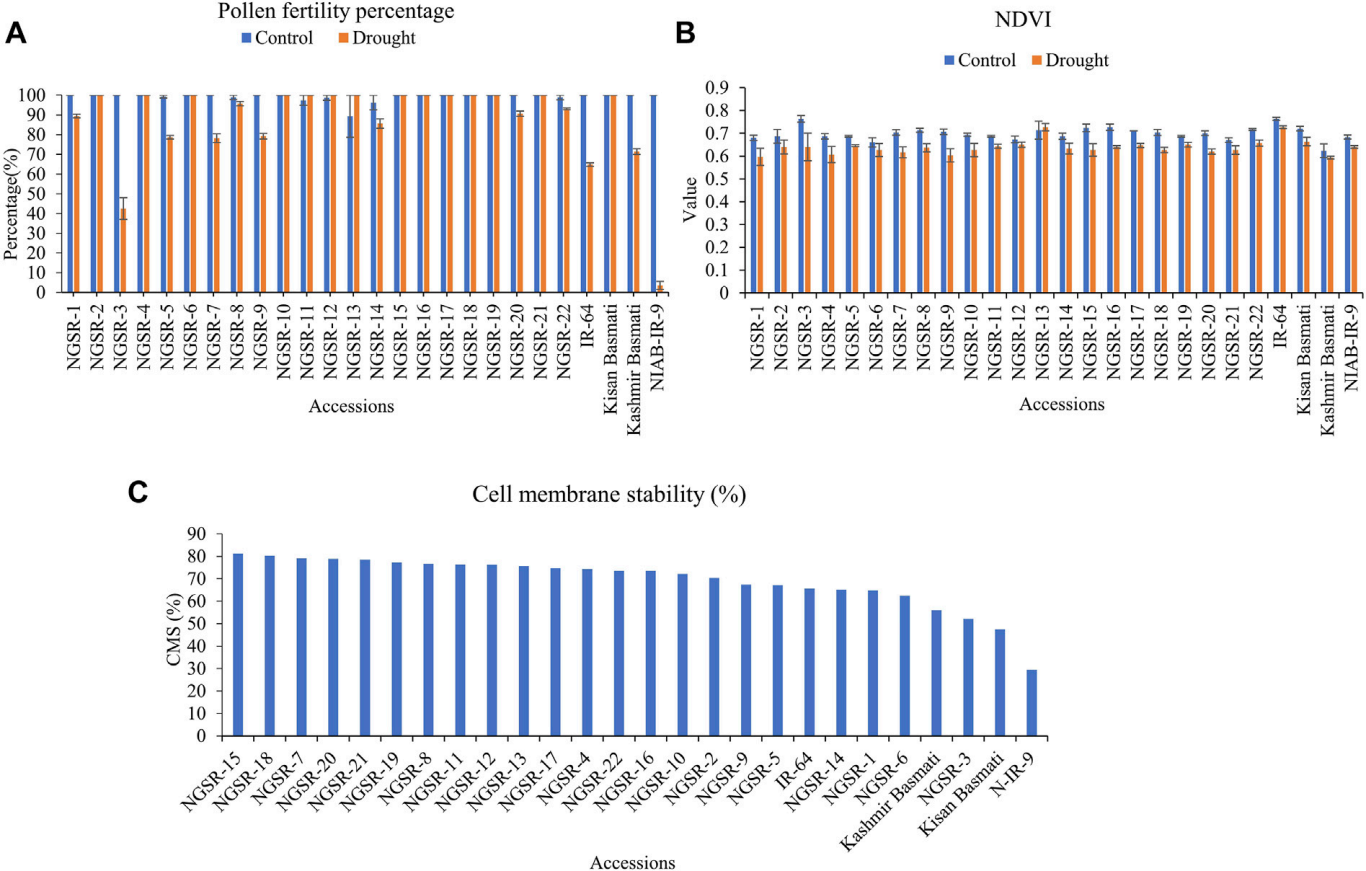
Effect of drought stress on **(A)** pollen fertility percentage, **(B)** normalized difference vegetative index (NDVI), and **(C)** cell membrane stability (CMS). Values (except CMS) are means ± SD.

Pollen fertility is an important indicator of drought tolerance as it directly affects the seed setting and ultimately the grain yield. The microscopic analyses of potassium iodide (I_2-_KI)-stained anthers revealed significant differences in PFPs between tolerant and sensitive genotypes. Overall, the GSR lines maintained higher PFPs under drought stress compared with non-GSR checks (Figure 5). While most of the GSR lines showed completely fertile pollens under drought, a higher sterility up to 57.1% was recorded in NGSR-3 (Figures 5 and 6A). In contrast, check varieties except the Kissan Basmati showed lower PFP compared with GSR under drought where NIAB-IR-9 showed only a 3.6% PFP (Figure 6A). These findings suggest that PFP could be a good indicator for drought tolerance in rice.

### Normalized difference vegetative index

NDVI has recently emerged as an indicator of plant health. We observed a considerable decrease in the value of NDVI under drought stress for GSR and check varieties (Figure 6B). The genotypes NGSR-13, IR-64, and Kissan Basmati showed the highest NDVIs (>0.7) under drought. Among GSR lines, the maximum NDVI (0.7) was reported in NGSR-13, whereas the minimum (0.59) was reported in NGSR-1. Similarly, among the check varities, the highest NDVI was depicted by IR-64 (0.72), whereas it was minimum by Kashmir Basmati (0.6) (Figure 6B). Since drought often causes leaf yellowing in plants, the reduced NDVI values under drought could be associated with yellow leaves.

### Cell membrane stability

CMS indicates the stress tolerance ability of plant cells. Again, GSR lines showed higher CMS% than non-GSR, where NGSR-15 showed the highest (81.1%) CMS followed by NGSR-18 (Figure 6C), while the lowest was measured in NIAB-IR-9 (29.5%).

### Correlation of grain yield with other agronomic traits

**FIGURE 7:**
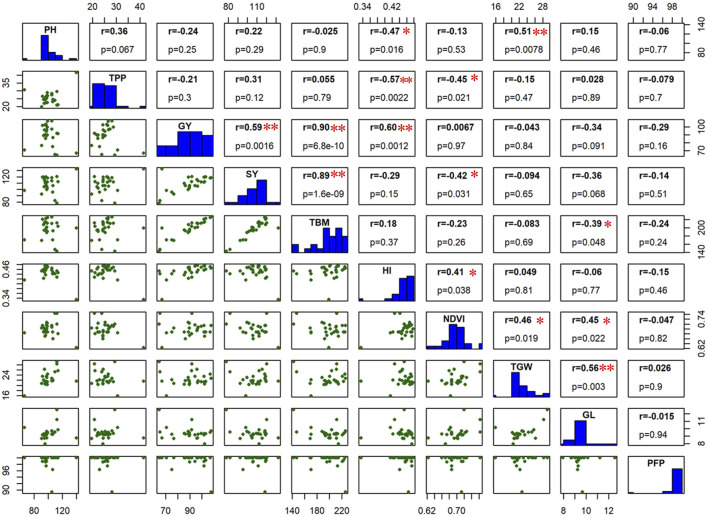
The scatter matrix below the histogram and correlation coefficient value with *p*-value above the histogram calculated from the means of all the studied traits under well-watered (WW) environmental condition. The *p*-values of all correlations were 0.05* and 0.01**.

Understanding the correlation of grain yield with other agronomic and physiological traits is of prime importance as it helps to identify certain prebreeding traits that could be best indicators of grain yield. Under WW environment, GY has shown a significant positive correlation with SY (r = 0.59**), TBM (r = 0.90**), and HI (r = 0.60**) (Figure 7). In addition, a significant negative correlation was found for TPP with HI (r = −0.57**), which suggests the importance of optimum number of tillers for better HI and GY.

**FIGURE 8:**
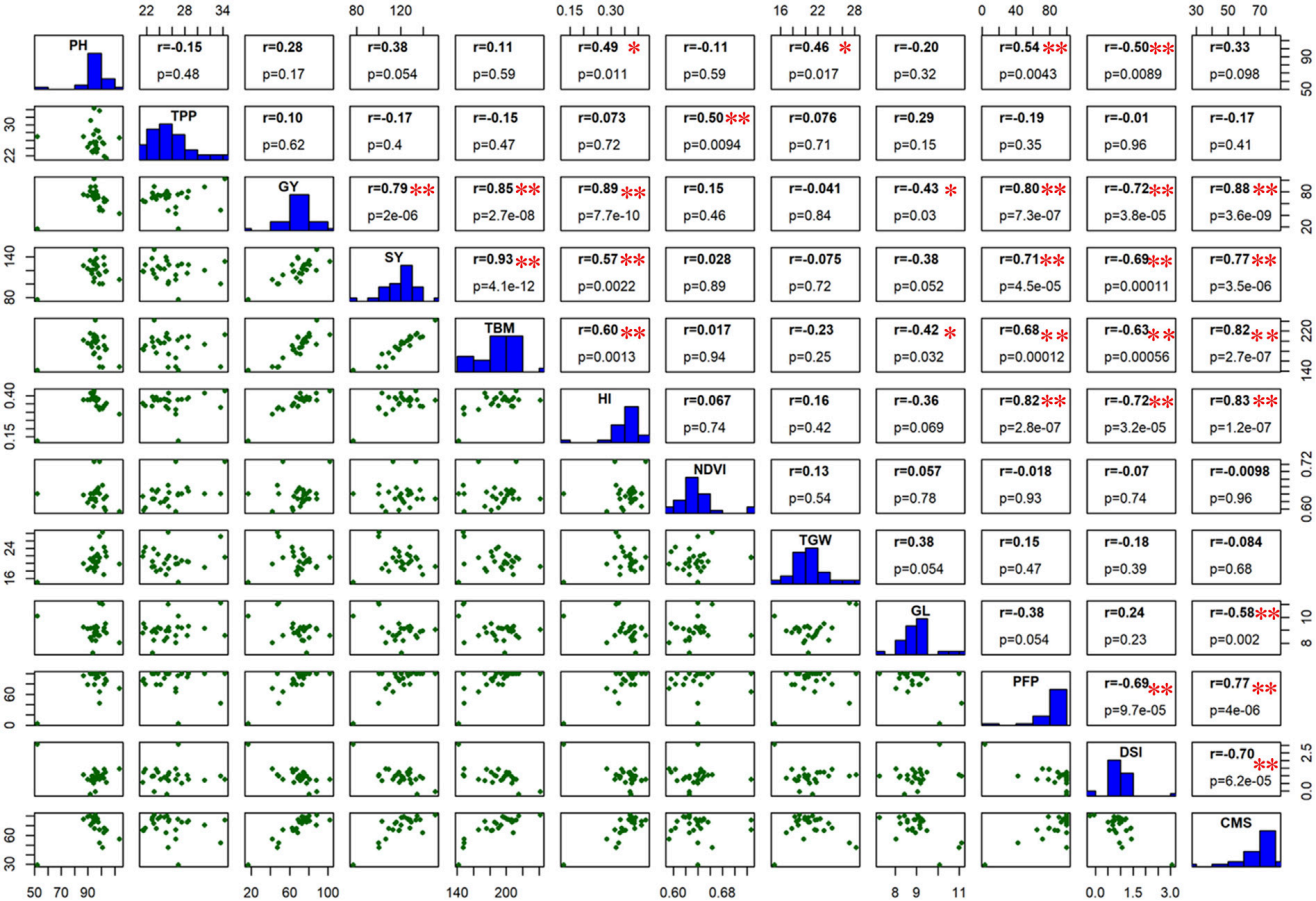
The scatter matrix below the histogram and correlation coefficient value with *p*-value above the histogram calculated from the means of all the studied traits under drought stress condition. The *p*-values of all correlations were 0.05* and 0.01**.

Under drought stress, GY has shown a significant positive correlation with HI (r = 0.89***), CMS (r = 0.88***), TBM (r = 0.85***), PFP (r = 0.80***), and SY (r = 0.79***), while a significant negative correlation was found with DSI (r = −0.72***) and GL (r = −0.43*) (Figure 8). In addition to GY, PFP has shown a significant positive correlation with HI (r = 0.82**), CMS (r = 0.77**), SY (r = 0.71**), and TBM (r = 0.68**), while a significant negative correlation of PFP was observed with DSI (r = −0.69**). Notably, DSI had significant negative correlations with GY (r = −0.72**), HI (r = −0.72**), CMS (r = −0.70**), SY (r = −0.69**), PFP (r = −0.69**), TBM (r = −0.63**), and PH (r = −0.50**), which suggest the importance of DSI being an important indicator of drought susceptibility in rice (Figure 7). These findings revealed important agronomic and physiological traits to be considered as reliable selection criteria for screening rice germplasm against drought tolerance.

### Expression analysis of drought-related genes

**FIGURE 9:**
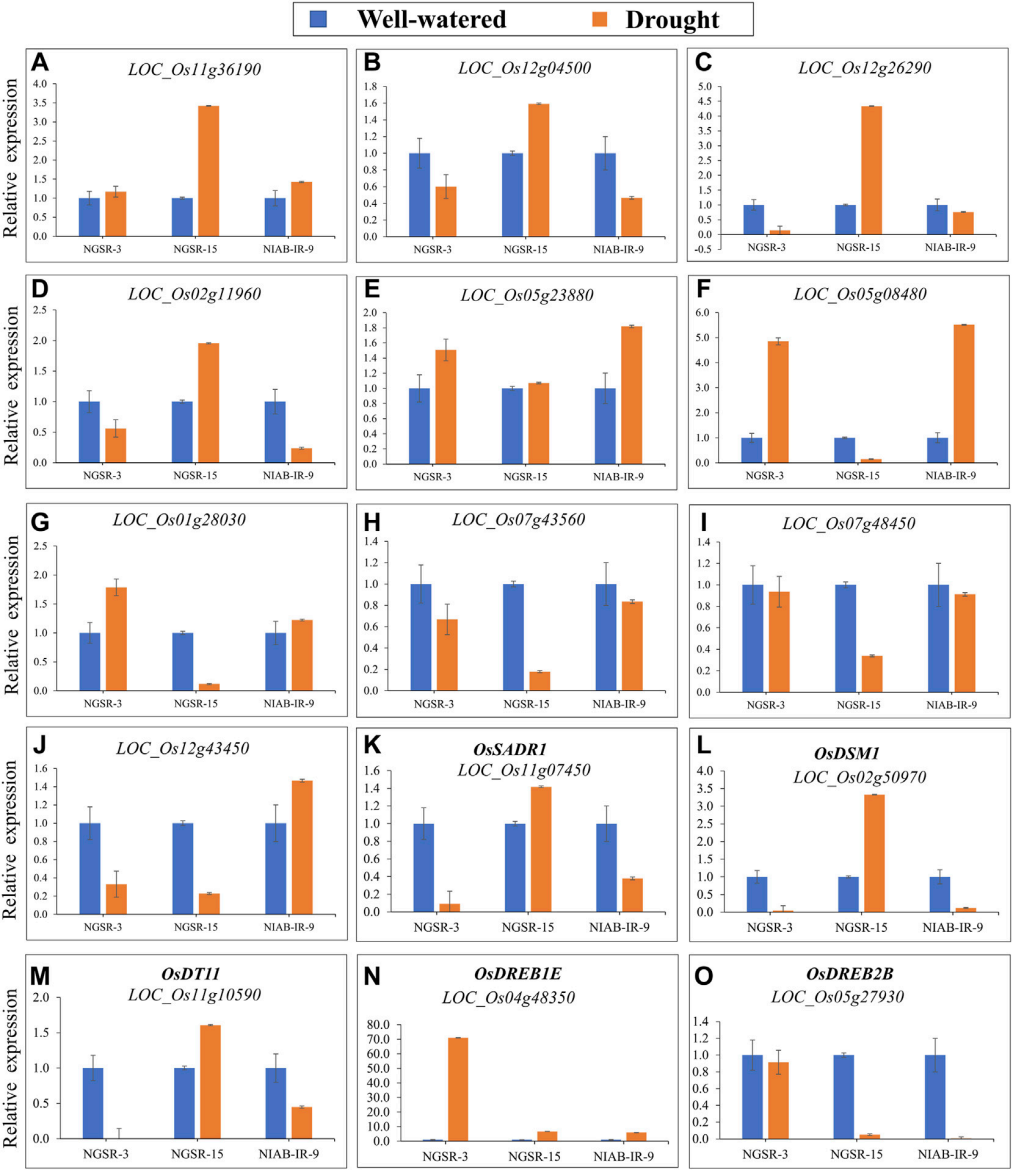
(qRT)-PCR analysis of drought-responsive genes in NGSR-3, NGSR-15, and NIAB-IR-9 revealed the relative expression in terms of fold change (log2FC). Young panicle tissues (∼1.5 cm) of three selected genotypes were employed in this analysis. Rice actin gene (*OsACT1*) was the internal control gene. Values of three biological replicates (*n* = 3) were expressed as mean ± SD.

**FIGURE 10:**
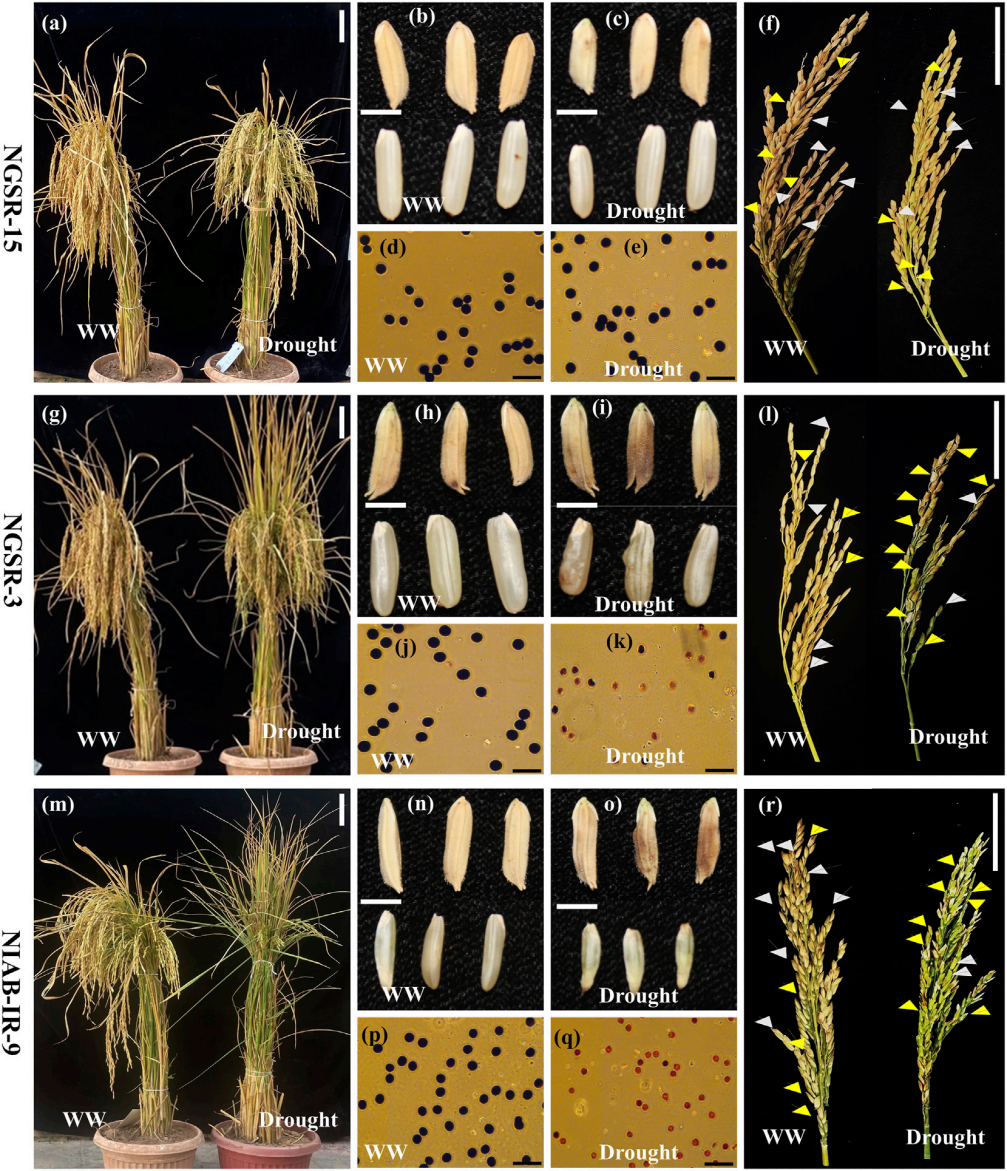
Phenotypic comparison of plants, grain length, and shape (husked and de-husked), pollen viability, and panicle fertility of NGSR-15, NGSR-3, and NIAB-IR-9 under well-watered (WW) and drought stress. Abbreviations: F, fertile spikelets; S, sterile spikelets. White arrow heads denote fertile spikelets and yellow arrow heads denote sterile spikelets. Spikelets with open tips represent sterile spikelets with no seed set. Scale bars are approximately 15 cm **(A,G,** **M)**, 5 mm **(B,C,H,I,N,** **O)**, 5 cm **(F,L,** **R)**, and 50 µm **(D,E,J,K,P,** **Q)**.

To see the role of drought responsive genes in drought tolerance of GSR, we analyzed the expression pattern in selected drought-tolerant and drought-sensitive genotypes using quantitative real-time PCR (Figure 9). The genotype NGSR-15 was selected as drought tolerant, and NGSR-3 and NIAB-IR-9 were chosen as drought-susceptible genotypes (Figure 10). Ten differentially expressed genes (DEGs) were selected for qRT-PCR analysis from the comparative transcriptome dataset between drought-sensitive (HHZ) and -tolerant (H471) genotypes (Huang et al., 2014). These genes have not been studied previously for their role in drought tolerance, except the transcriptome analysis. In addition, we analyzed the expression of five previously characterized genes for drought tolerance in rice (*OsSADRI*, *OsDSM1*, *OsDT11*, *OsDREB1E*, and *OsDREB2B*).

Our results indicated that four genes, namely, *LOC_Os11g36190* (receptor kinase), *LOC_Os12g04500* (response regulator receiver domain-containing protein), *LOC_Os12g26290* (alpha-DOX2), and *LOC_Os02g11960* (ABC transporter, ATP-binding protein), were upregulated in drought-tolerant genotypes (NGSR-15) and downregulated in drought-sensitive genotypes (NGSR-3 and NIAB-IR-9) (Figure 9). This suggest that these genes may positively regulate drought tolerance in rice. Three genes, namely, *LOC_Os05g23880* (lipoxygenase), *LOC_Os05g08480* (cytokinin-O-glucosyltransferase 1), and *LOC_Os01g28030* (peroxidase precursor), were downregulated in NGSR-15, while they were upregulated in drought-sensitive genotypes (NGSR-3 and NIAB-IR-9), suggesting a negative regulation for drought tolerance (Figure 9). Two genes, *LOC_Os07g43560* (protein kinase-like) and *LOC_Os07g48450* (no apical meristem protein), were downregulated under drought stress in all genotypes and, thus, may not be related to drought tolerance (Figure 9). The gene *LOC_Os12g43450* (thaumatin family domain-containing protein) was upregulated in NIAB-IR-9, while it was downregulated in both NGSR-3 and NGSR-15, suggesting that this gene might be related to nonGSR rice. These results revealed a differential expression pattern of genes among drought-tolerant and -sensitive genotypes and, thus, could be employed for molecular identification of drought-tolerant rice genotypes at large scale. Notably, we observed an increased expression of previously known drought tolerance-related genes (*OsSADRI*, *OsDSM1*, and *OsDT11*) in NGSR-15, while the opposite was observed for NGSR-3 and NIAB-IR-9, which clearly indicated the role of these genes in drought tolerance. In addition, *OsDREB1E* has shown a sharp increase in expression under drought stress in all the three genotypes, while *OsDREB2B* has shown a sharp decrease in expression under drought stress in NGSR-15 and NIAB-IR-9 (Figures 9N, O). This suggest that DREB genes are probably not directly involved in drought tolerance in these tested genotypes, and their expression is modulated by some unknown genetic factors.

## Discussion

Rice being a prime diet of 50% of the global population and the staple food of many countries is an important grain crop. However, growing rice requires high delta of water where limited water conditions affect its growth and grain yield. Water stress at anthesis directly affects seed setting and grain filling and, ultimately, the grain yield (Shokat et al., 2020a; Shokat et al., 2020b). Green super rice (GSR) was developed by combining the best global germplasm and has the potential to maintain the optimum grain yield under different stress conditions (Jewel et al., 2019). Moreover, this germplasm has never been evaluated for pre-anthesis drought stress. In the current experiment, 22 GSR genotypes and four local lines of Pakistan were used to understand the mechanism of yield reduction at pre-anthesis stages of drought stress. This germplasm was characterized for different agrophysiological traits, and then the most diverse genotypes were further evaluated by novel drought-responsive genes. Yield-related traits are important indications of final grain yield (Zafar et al., 2020a; Waqas et al., 2021). Studies reported that plant genotypes that maintained higher plant biomass under drought stress conditions often maintain higher grain number or weight and ultimately the grain yield (Shokat et al., 2021). In the current study, we identified NGSR-15 as a drought-tolerant line as it maintained higher CMS, PFP, TBM, and GY. In contrast, NGSR-3 and NIAB-IR-9 were ranked as drought-sensitive lines since they showed significant reductions in GY probably due to reduced CMS, PFP, and TBM. A phenotypic presentation of the performance of these genotypes under drought stress is shown in Figure 10. Higher biomass is usually linked with higher photosynthetic rate of the genotypes (Morinaka et al., 2006). Our results indicate that biomass partitioning toward grain filling was limited due to flowering stage drought stress, and there could be a possibility that GSR-15 has maintained a higher grain yield due to better seed setting under moisture stress conditions. To explain the possible mechanism of higher and lower grain yield for the genotype GSR-15 and NIAB-IR-9, respectively, we associated yield data with the few parameters of physiology to understand the physiological basis of yield reduction at flowering stage drought stress.

DSI indicates the extent of susceptibility by drought stress in terms of economically important traits particularly the grain yield. In this study, genotypes NGSR-15 and NGSR-18 showed the lowest susceptibility with values of −0.04 and −0.2, respectively, whereas NIAB-IR-9 (check) showed the highest DSI value of 3.6 (Figure 4). Under drought stress, permeability of membranes and leakage of ions occur from the weak or unstable membranes (Bajji et al., 2002). Likewise, seed setting is dependent on the viability of pollen, while limited water availability at the flowering stage can cause pollen abortion in sensitive genotypes (Mehri et al., 2020). In contrast, plant genotypes that show better cell membrane stability (CMS) or pollen fertility could perform better under flowering stage drought stress. In the current experiment, better cell membrane stability and pollen fertile percentage (PFP) was exhibited by the genotype NGSR-15, while the lowest values were recorded for NGSR-3 and NIAB-IR-9 indicating the physiological basis of drought tolerance and drought susceptibility of these genotypes respectively. A correlation and PCA was drawn to test the significance of these parameters in relation to yield and yield-related traits, and we found a strong significant and positive correlation of CMS and PFP with grain yield (Figure 1B). In contrast, association of grain yield was significant but negatively associated with DSI (Figure 1B) indicating that these traits could be selected as prebreeding traits for flowering stage drought stress in rice. To understand the molecular basis of drought tolerance, these three genotypes were further tested through gene expression.

Stress conditions change the expressions of the stress-induced regulatory or effector genes, which are usually involved in the regulation of normal processes of the plants (Ouyang et al., 2010). We investigated different categories of DEGs, controlling drought tolerance and sensitivity by up-/downregulation of DEGs. Furthermore, this analysis relied on two GSR genotypes and one locally developed genotype, NIAB-IR-9, in order to provide an accurate estimate of expression by comparing GSRs with traditional cultivars under flowering stage drought stress. In agreement with published literature, our expression results suggest the involvement of DEGs in drought tolerance or sensitivity (Chen et al., 2009; Narsai et al., 2013; Zhang et al., 2015b). Leucine-rich RLKs, play a key role in the regulation of plant growth under various abiotic stresses, and gene *LOC_Os11g36190* (a leucine-rich receptor-like kinase) is predicted to be upregulated for bacterial leaf blight in rice (Zhang et al., 2015a; Ahsan et al., 2019). *LOC_Os12g04500* and *LOC_Os12g26290* are also reported as the core of the jasmonic acid (JA) signaling pathway, and in the current experiment, expression of these two genes was increased significantly under prolonged drought period. Moreover, JA signaling genes are also reported to be involved under critical phases of drought stress (Du et al., 2007). We found that four genes, i.e., *LOC_Os11g36190*, *LOC_*Os12g04500, LOC_Os12g26290, and LOC_Os02g119600, were upregulated in drought-tolerant genotypes (NGSR-15) and downregulated in drought-sensitive genotypes (NGSR-3 and NIAB-IR-9) indicating their positive relationship with drought tolerance. Likewise, an increased expression of previously known drought tolerance-related genes (*OsSADRI*, *OsDSM1*, and *OsDT11*) (Ning et al., 2009; Li et al., 2017; Park et al., 2018) was observed in NGSR-15, while an opposite trend was observed for NGSR-3 and NIAB-IR-9. This change in expression in the tested genes could be due to a sequence variation in their promoter region or mutation in a major upstream regulator, which is currently unknown to us. Apart from gene expression, these genotypes also showed a contrast for PFP, CMS, and DSI along with clear differences in grain yield suggesting their role in terminal-stage drought tolerance. Drought-responsive element-binding proteins (DREBs) are known to play important roles in abiotic stresses especially drought (Chen et al., 2008). Interestingly, in our study, expression of the DREB gene was either increased in both tolerant and sensitive genotypes, or decreased under drought stress (Figures 9M, O). This suggests that DREB genes are probably not directly involved in drought tolerance in these tested genotypes, and their expression is modulated by some unknown genetic factors.

## Conclusion

Through this study, we identified molecular and physiological basis of higher grain yield at the flowering stage drought stress and the role of novel drought-responsive genes in drought tolerance. Importantly, various morphophysiological traits (PFP,CMS, DSI, and HI) had strong association with drought-responsive genes, and ultimately, the grain yield indicating these parameters could be used as prebreeding traits for drought tolerance. Our results also indicate that genotype NGSR-15 was the most drought tolerant, while NGSR-3 and NIAB-IR-9 were the most sensitive genotypes. These genotypes can further be used to improve rice yield under drought stress; however, in-depth mechanism is required to confirm our findings.

## Data Availability

The original contributions presented in the study are included in the article/Supplementary Material, further inquiries can be directed to the corresponding authors.
